# Supplementary material for: IκBα controls dormancy in hematopoietic stem cells via retinoic acid during embryonic development
Source: Nat Commun. 2024 Jun 1;15:4673. doi: 10.1038/s41467-024-48854-5 (PMC11144194; doi:10.1038/s41467-024-48854-5)
Supplement: Supplementary file 1 — Supplementary Information [file 41467_2024_48854_MOESM1_ESM.pdf]

Supplementary Figure S1

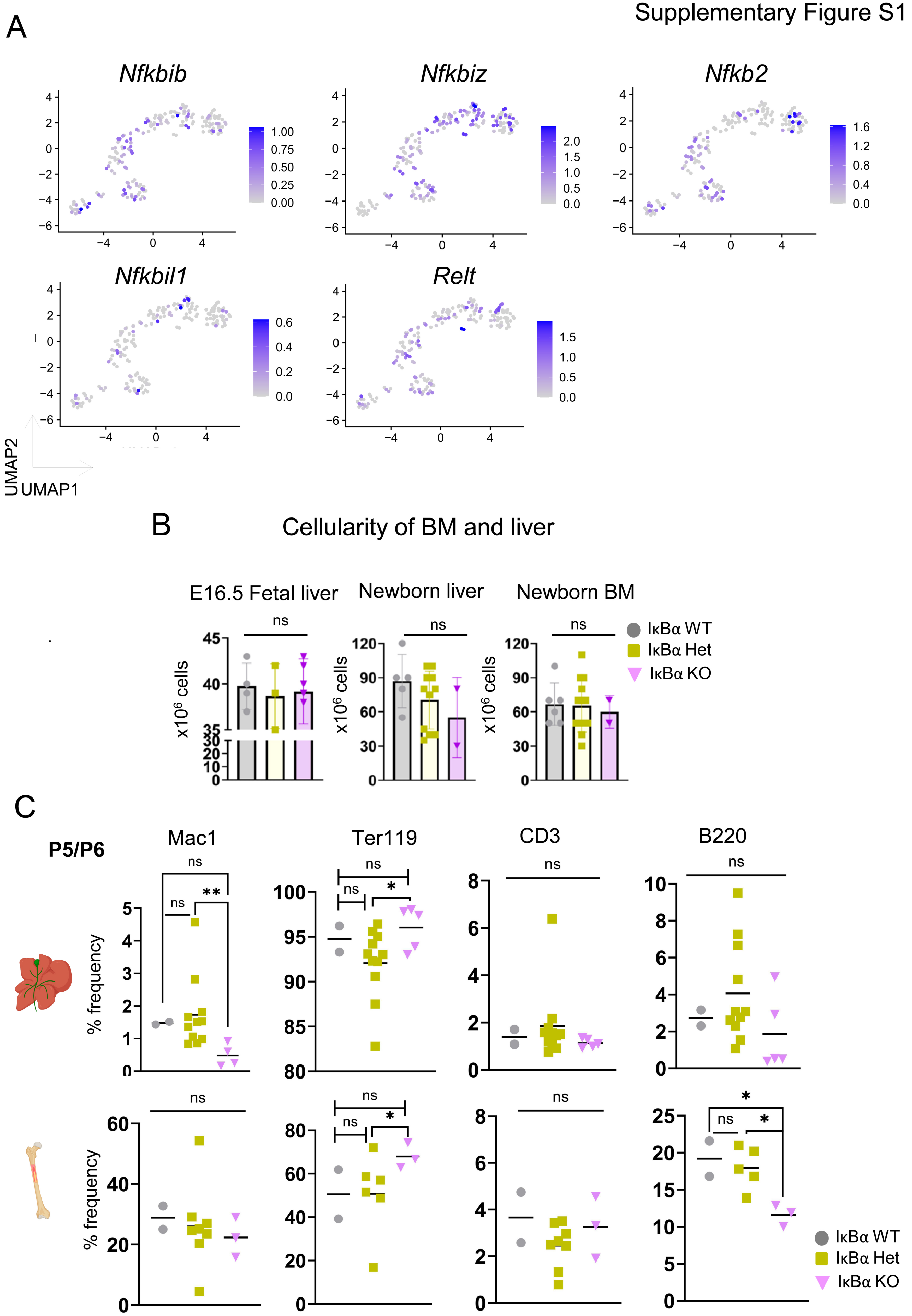

Suppl Figure S1: NF-κB signaling elements present in HSCs (**A**) Representation of gene expression levels of NF-κB signaling elements across the UMAP of single cell RNA-seq data set from Zhou et al, 2016. (**B**) Scatter plot with the total numbers of E16.5 fetal liver (WT n=4, HET n=3, KO n=5) and P5/6 liver (WT n=5, HET n=11, KO n=2) and femurs (WT n=6, HET n=13, KO n=2) after red blood cell lysis obtained from n= 9 WT, 14 het and 7 IκBα KO in 3 independent experiments. Statistical test: one-way ANOVA (ns p-value > 0.05). Bars indicate mean values and error bars refer to +/- standard deviation. (**C**) Individual values for the frequency of Ter119 (erythroid cells) B220 (B-cells), CD4/8 (T cells) and Gr1/Mac1 (myeloid cells) in fetal liver (top) and bone marrow (bottom) of newborn (P5/6) IκBα WT, Het and KO obtained from n=18 pups in 2 independent experiments. Statistical test: unpaired two-tailed Dunn's non-parametric all-pairs comparison test after Kruskal-Wallis test (**p-value < 0.01 * p-value < 0.05, ns p-value>0.05). Horizontal bars indicate mean values. Source data are provided as a Source Data file. Panels C was created with BioRender.com released under a Creative Commons Attribution-NonCommercial-NoDerivs 4.0 International license.

Supplementary Figure S2

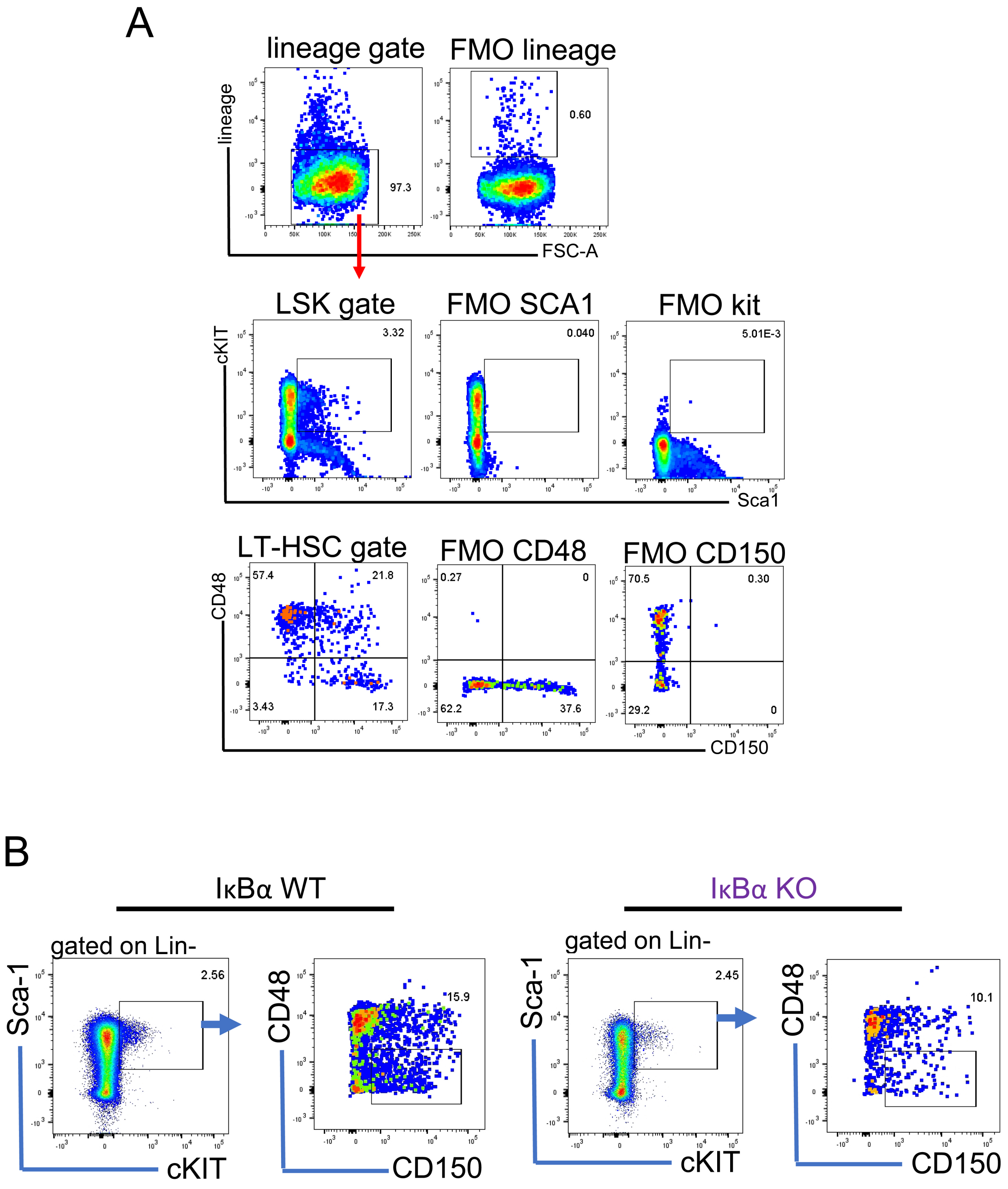

Suppl Figure S2: Blood lineage profiling of *IκBα* KO adult, newborn, and fetal liver hematopoiesis (**A**) Fluorescence minus one (FMO) control panel for the LT-HSC determination FACS panel. (**B**) Representative FACS plots gating strategy for LSK (lin-SCA1+cKIT+) and LT-HSC (LSKCD48-CD150+) of *IκBα* WT, Het, and KO bone marrow and (fetal) liver.

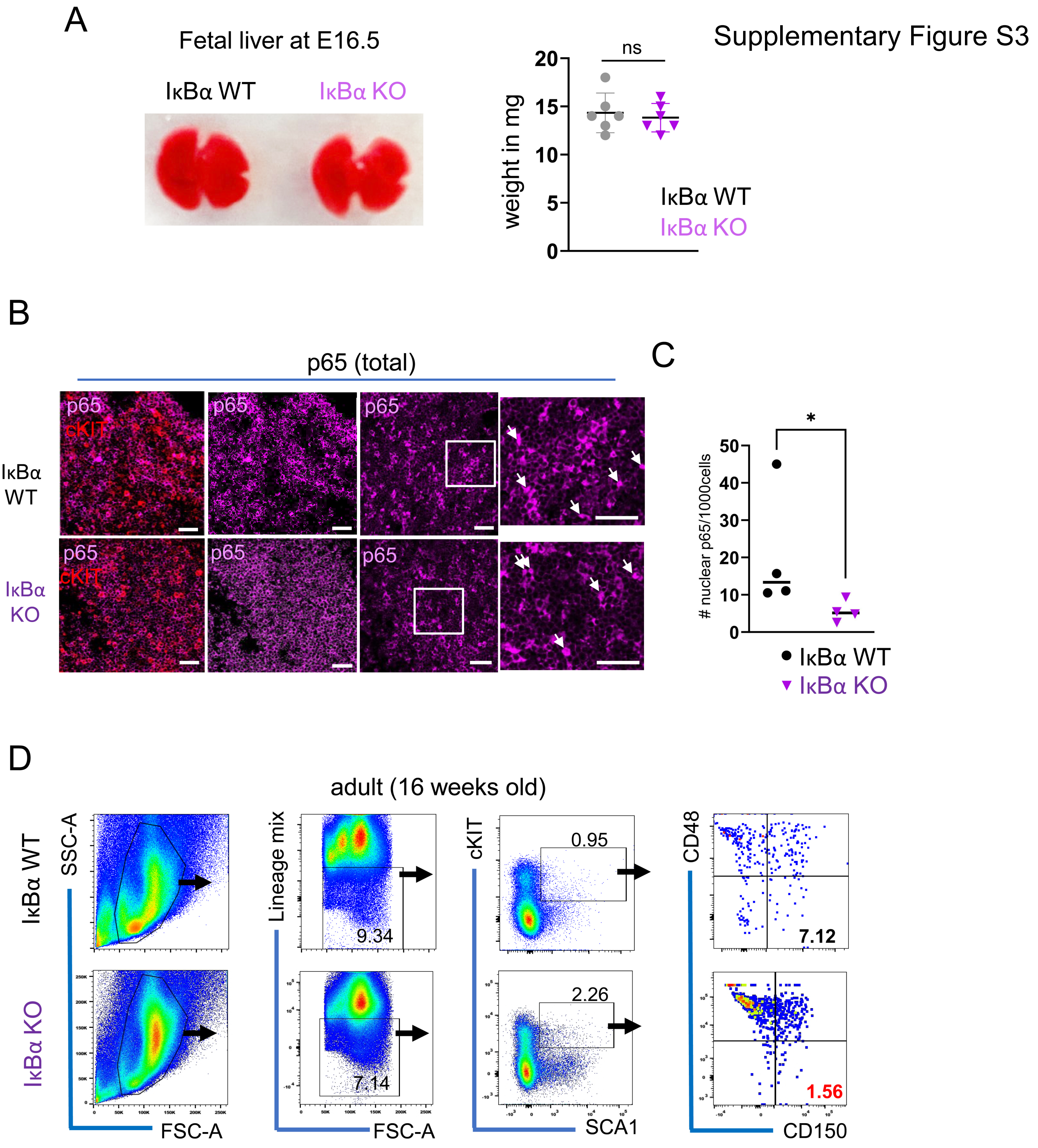

Suppl Figure S3: P65 status in E14.5 Fetal liver and Stem cell profiling of *IκBα* KO adult (A) Representative brightfield image and weight in mg of *IκBα* WT (n=6) and KO (n=6) E16.5 fetal liver obtained per n= 6 embryos per genotype. Images were taken on an IphonePro13. Statistical test: unpaired one-tailed t-test with Welch correction (ns p-value > 0.05). The horizontal bars indicate mean values and error bars refer to +/- standard deviation. **(B)** Representative images of IHC on E14.5 Fetal liver section of *IκBα* WT and KO for p65 (magenta), c-KIT (red) and DAPI (blue). n=4 sections from 2 different embryos. Scale bar: 50μm. Images were taken using a SPE (Leica) with a 20x oil lens and processed using ImageJ. **(C)** Quantification of nuclear p65 positive cells normalized to 1000 cells. Total counts were performed with ImageJ and nuclear p65 cells were determined manually. Statistical test: unpaired one-tailed Mann Whitney U test (* p-value < 0.05). The horizontal bars indicate mean values. **(D)** Representative FACS plots for the frequency of LSK and LT-HSC in one adult (3 months old) bone marrow of *IκBα* WT and KO. n=1 *IκBα* het and 1 *IκBα* KO in one experiment. Source data are provided as a Source Data file.

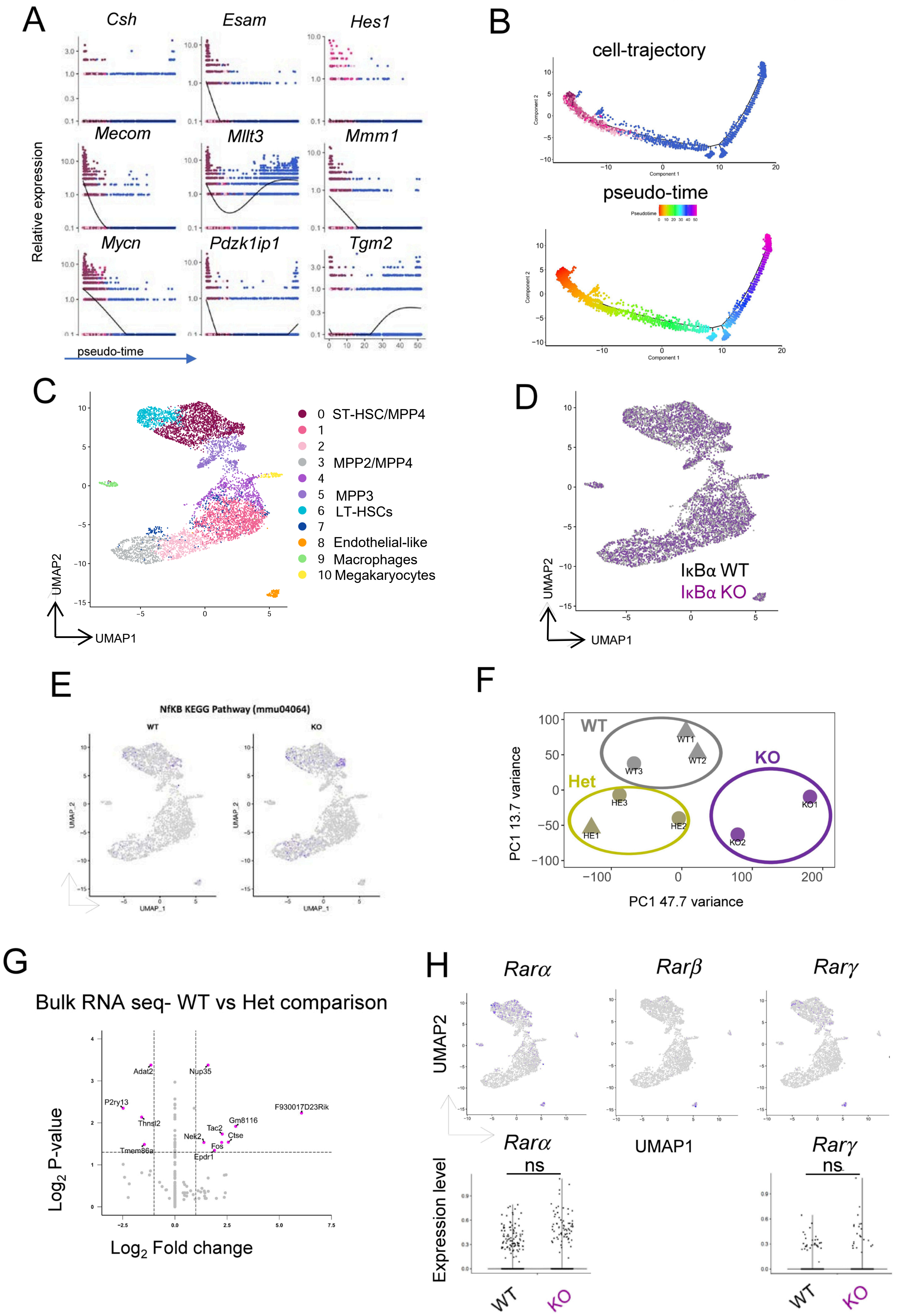
A
Csh
Esam
Hes1
Mecom
Mllt3
Mmm1
Mycn
Pdzk1ip1
Tgm2
Relative expression
pseudo-time
B
cell-trajectory
Component 1
Component 2
pseudo-time
Pseudotime
C
0 ST-HSC/MPP4
1
2
3 MPP2/MPP4
4
5 MPP3
6 LT-HSCs
7
8 Endothelial-like
9 Macrophages
10 Megakaryocytes
UMAP2
UMAP1
D
IκBα WT
IκBα KO
UMAP2
UMAP1
E
NfKB KEGG Pathway (mmu04064)
WT
KO
UMAP_2
UMAP_1
F
WT
WT1
WT2
WT3
Het
HE1
HE2
HE3
KO
KO1
KO2
PC1 13.7 variance
PC1 47.7 variance
G
Bulk RNA seq- WT vs Het comparison
Adat2
Nup35
P2ry13
Thnsl2
F930017D23Rik
Gm8116
Tac2
Ctse
Nek2
Fos
Tmem86a
Epdr1
Log2 P-value
Log2 Fold change
H
Rarα
Rarβ
Rarγ
UMAP2
UMAP1
Rarα
ns
Rarγ
ns
Expression level
WT
KO
WT
KO

Suppl Figure S4: Molecular characteristics of E14.5 *IκBα* KO LT-HSCs (**A**) Scatter plot of gene expression levels of LT-HSC defining genes (Rodriguez-Fraticelli et al, 2018) for individually RNA-sequenced *IκBα* WT and KO cells. (**B**) Trajectory/Pseudo-time analysis of all individually sequenced cells. (**C**) UMAP based clustering of all the single cells sequenced, and their assigned cell identity based on published data sets. (**D**) UMAP highlighting the *IκBα* genotype (WT or KO) across all sequenced cells. (**E**) UMAP with cumulative gene expression levels of the NF-κB signaling signature (mmu04064), separated by *IκBα* genotype (WT or KO). (**F**) Principal Component clustering (PCA) of the LT-HSC RNA-sequencing samples from 3 WT, 3 Het and 2 KO for *IκBα* . (circle: female embryos, triangle: male embryos). (**G**) Scatter plot of DEG and their p-values for *IκBα* WT compared to HET. (**H**) UMAP (top) and scatter plot (bottom) for the gene expression levels of the retinoic acid receptors, *Rarα*, *Rarβ* and *Rarγ* in single cells and separated for *IκBα* genotype (WT or KO). P-value determined with two tailed unpaired t-test (ns p-value > 0.05).

Supplementary Figure S5

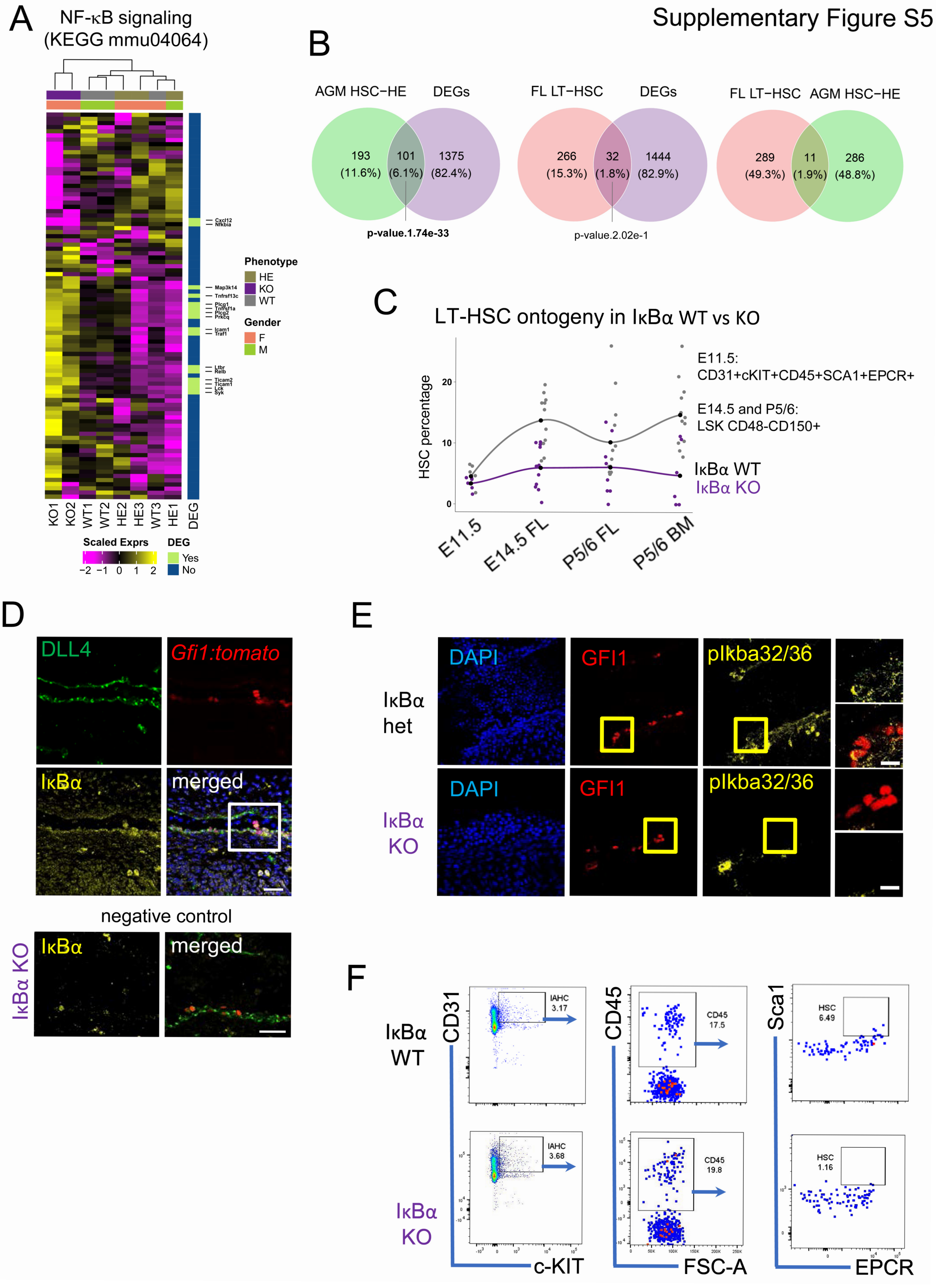

Suppl Figure S5: *IκBα* KO AGMs have reduced number of GFI1/EPCR positive cells and the LT-HSCs in the fetal liver retain AGM-associated gene signature (**A**) Heatmap showing the expression levels of NF-κB signaling genes (KEGG Pathway ID mmu04064) from *IκBα* WT/Het and KO E14.5 LT-HSCs RNAseq samples (**B**) enn diagrams of overlapping genes in the gene sets of the top 300 genes expressed in AGM HSC/HE (green), DEG of the RNA-seq of *IκBα* WT/Het and KO LT-HSCs (magenta, FDR adj pval < 0.05 and absolute shrunken log2 fold change >1), and the top 300 genes exclusively expressed in E14.5 fetal liver HSCs when compared to the bone marrow LT-HSCs (Manesia et al, 2017). Associated p-value determined with hypergeometric test (upper-tail) considering a total set of 1,472 DEGs and a background of 16k genes for both signatures. (**C**) Chart showing the HSC percentage, i.e., of SCA1-EPCR (E11.5) or CD48-CD150+ (E14.5 FL and P5/6 newborn fetal liver and bone marrow) from previous gate as determined in Figure 2Bii and 3Gii. (**D**) Representative image of IHC on E11.5 (43-45s) sagittal AGM section for endothelial cells (DLL4, green) and *IκBα* (yellow) and DAPI (blue) on *Gfi1:tomato* (red) transgenic embryos. Scale bar: 50µm. n= 4 WT and 5 KO AGM 12µm sections derived from 2 embryos for each genotype. Images were taken using a SPE (Leica) with a 20x oil lens and processed using Imaris. **(E)** Representative image of IHC on E11.5 (43-45s) sagittal AGM section for and pIκBα Ser32/36 (yellow) and DAPI (blue) on *Gfi1:tomato* (red) transgenic embryos. Scale bar: 50µm. n= 2 Het and 3 *IκBα* KO AGMs from 2 independent experiment (embryos). Images were taken using a SPE (Leica) with a 20x oil lens and processed using ImageJ. **(F)** Representative FACS plots for CD31+/cKIT+/CD45+/SCA1+/EPCR+ staining in E11.5 AGMs of *IκBα* WT/Het and KO. Source data are provided as a Source Data file.

Supplementary Figure S6

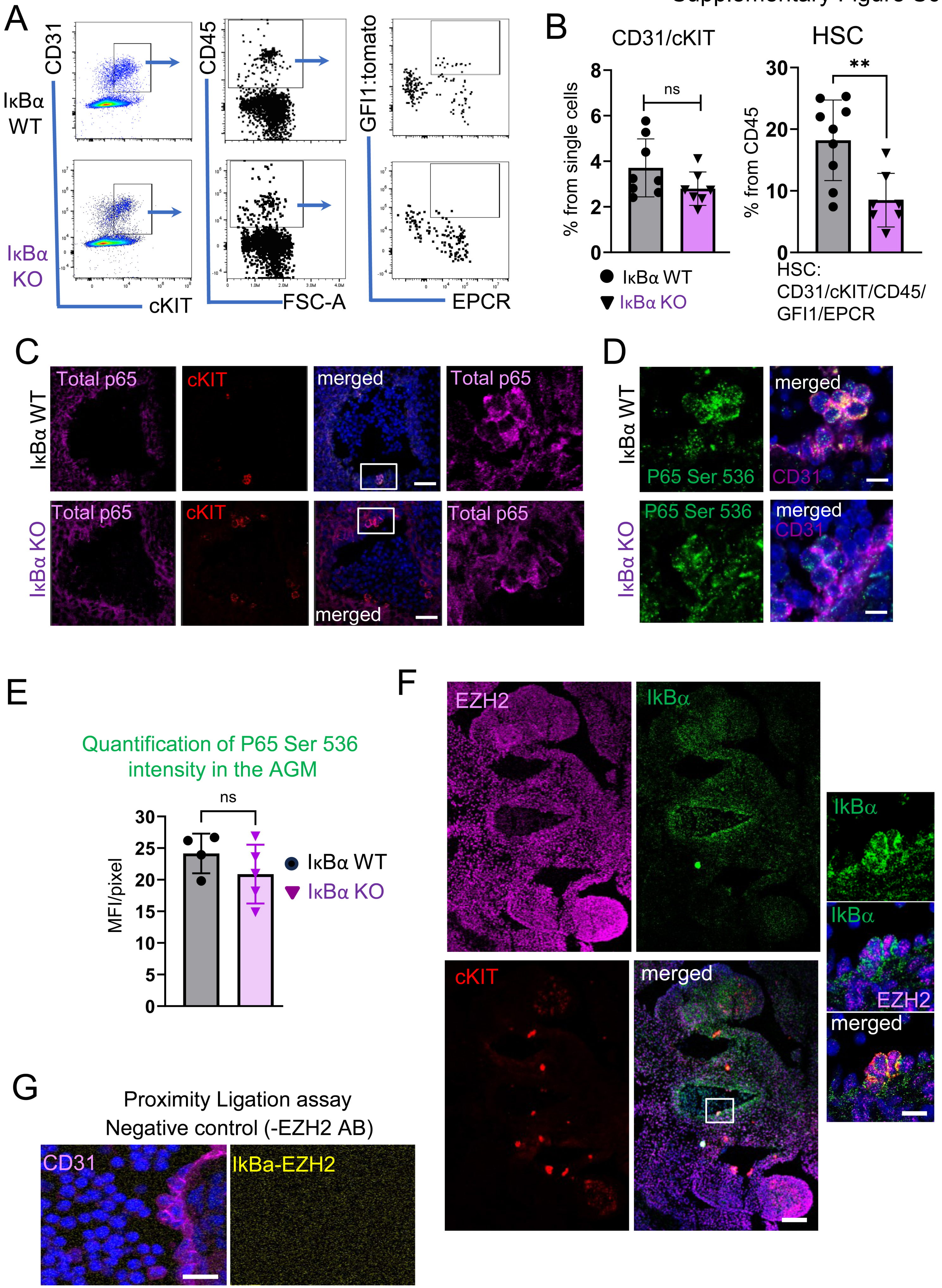

Suppl Figure S6: IHC of p65 and p65 Ser-536 *IκBα* T and KO AGMs and IHC for E H and *IκBα*. (**A**) Representative FACS plots for CD31/cKIT/CD45/GFI1/EPCR+ staining in E11.5 (43-47 somites) AGMs of *IκBα* WT/Het and KO. **(B)** Bar chart with percentage of CD31+/cKIT+/CD45+ and CD31+/cKIT+/CD45+/GFI1+/EPCR+ cells by FACS in E11.5 (42-46 somites) AGMs of *IκBα* WT/Het (n=8 and n=9, respectively) and KO (n=7 and n=7, respectively). Statistical test: Multiple linear regression model with Experiment and Genotype covariates, significance obtained from applying t-test to corresponding model coefficient estimate (** p-value <0.01, ns p-value >0.05). Bars indicate mean values and error bars refer to +/- standard deviation. **(C)** Representative image of IHC on E11.5 (43-45somites) AGM section of *IκBα* WT and KO for p65 (magenta), c-KIT (red) and DAPI (blue). Scale bar: 50µm. n=1 AGM section per genotype with 2 IAHC in *IκBα* WT and 3 IAHC in *IκBα* KO. Images were taken using a SPE (Leica) with a 20x oil lens and processed using Imaris. (**D**) Representative images of IHC on E11.5 (43-45 somites) AGM section of *IκBα* WT and KO for phospho-p65 Ser536 (green), CD31 (magenta) and DAPI (blue). Scale bar: 10µm. n= 4 WT and 6 KO AGM sections from 2 independent experiments (embryos). Images were taken using a SPE (Leica) with a 20x oil lens and processed using Imaris. (**E**) Mean fluorescence intensity (MFI) normalized to pixel count in the CD31 area (WT n=4, KO n=5). (IAHC locations are highlighted with a yellow circle (as detected by cKIT). The measurements were performed with ImageJ measure setting. Statistical test: unpaired one-tailed t-test with Welch correction (ns p-value > 0.05) (**F**) Representative image of IHC on E11.5 (43-45 somites) AGM section of *IκBα* WT for E H2 (magenta), *IκBα* (green), cKIT (red) and DAPI (blue). Scale bar: 100um and 10um (inlet). n=8 sections from 2 independent experiments (embryos). Images were taken using a SPE (Leica) with a 10x dry lens or 20x oil lens and processed using Imaris. (**G**) Negative control for PLA experiment. Sample without the primary E H2 antibody. E11.5 (43-45 somites) AGM section of PLA IκB /-E H2 (yellow), CD31 (magenta) and DAPI (blue). Scale bar: 25um. n=1 experiments. Images were taken using a Stellaris8 (Leica) with a 20x oil lens and processed using Imaris. Source data are provided as a Source Data file.

Supplementary Figure S7

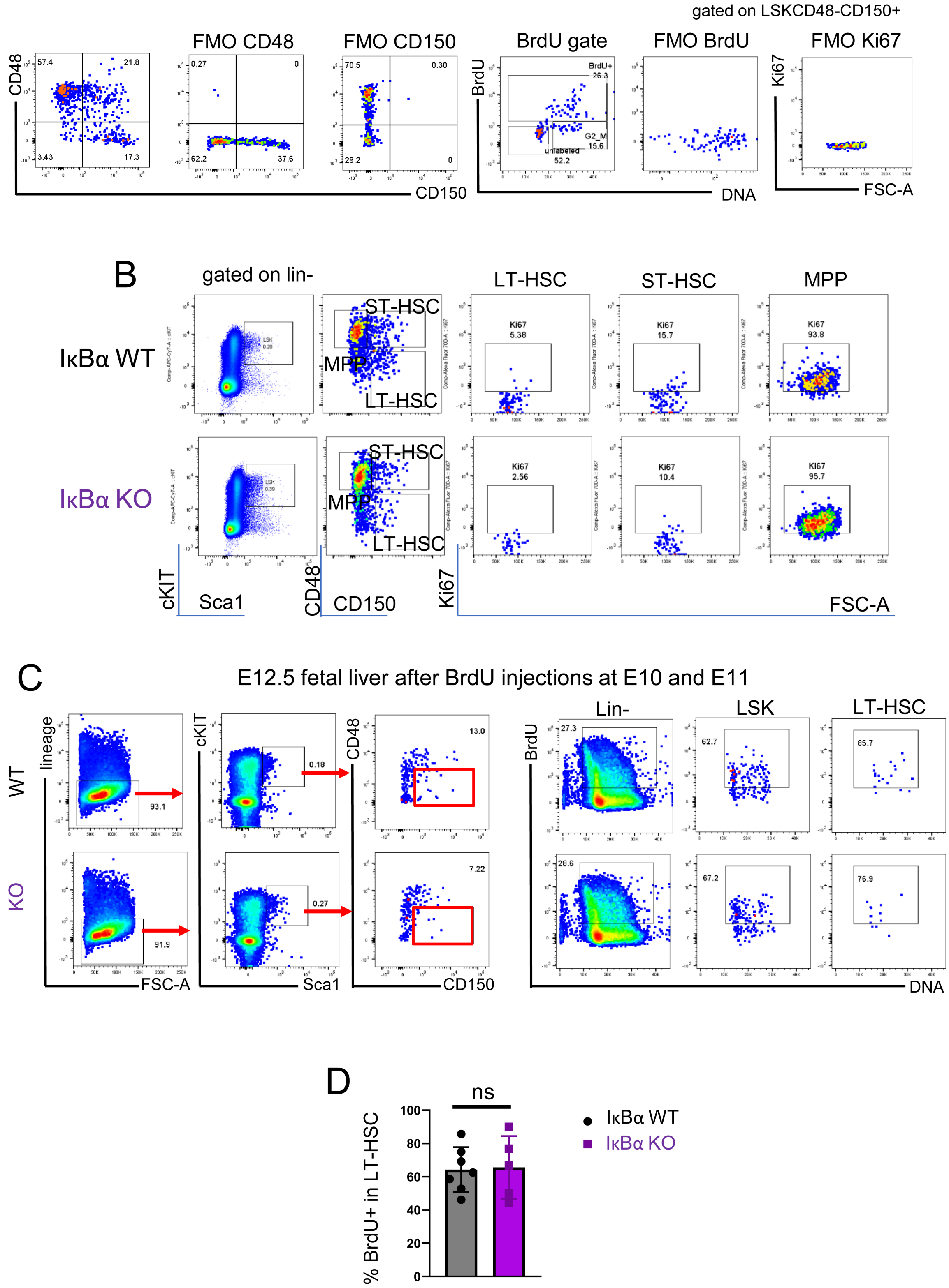

Suppl Figure S7: IκBα WT and KO LT-HSCs can be labeled in BrdU labeling experiment. (**A**) Fluorescence minus one (FMO) control for the BrdU or Ki67 gate in FACS analysis. (**B**) representative FACS plots of Ki67+ cells in MPP (multi-potent progenitors), ST-HSCs and LT-HSCs of E14.5 *IκBα* WT/Het and KO fetal liver samples. (**C**) exemplary FACS plots of BrdU+ cells in LT-HSCs of E12.5 *IκBα* WT/Het and KO fetal liver samples after two injections of 2mg of BrdU at E10 and E11. (**D**) Bar chart representation of the percentage of BrdU+ LT-HSCs of E12.5 *IκBα* WT/Het (n=7) and KO (n=5) fetal liver samples after two injections of 2mg of BrdU at E10 and E11 obtained from n= 12 in 2 independent experiments. Statistical test: unpaired one-tailed t-test with Welch correction (ns p-value > 0.05). Bars indicate mean values and error bars refer to +/- standard deviation. Source data are provided as a Source Data file.

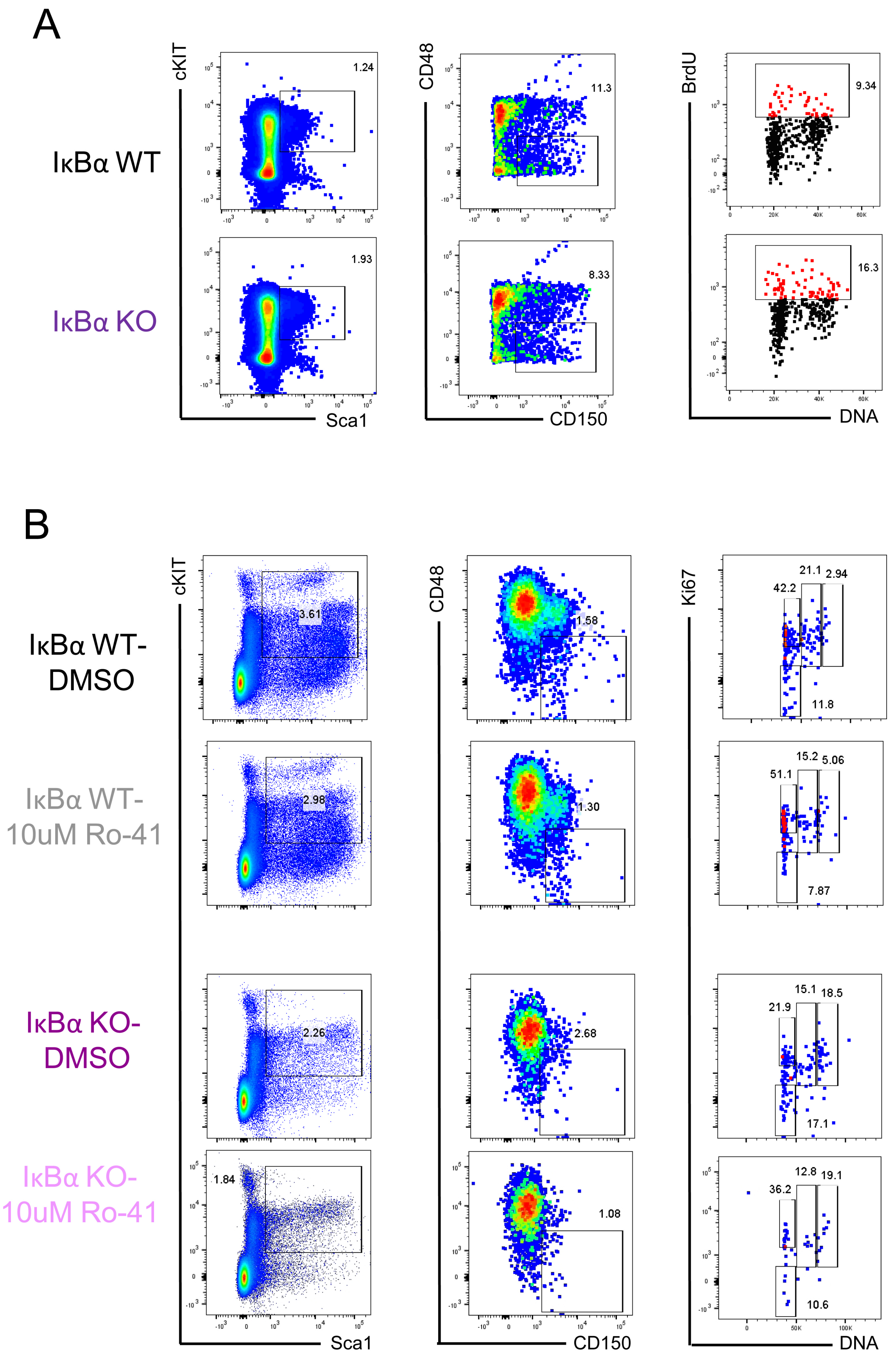

Suppl Figure S8: Representative FACS plots for BrdU+ cells and G0 determination in LT-HSCs (**A**) Re-presentative FACS plots of the percentage of BrdU+ cells within the E14.5 FL LT-HSC after *in vivo* labeling of E10.5 *IκBα* WT or KO embryos. (**B**) Exemplary FACS plot with the percentage of LT-HSC in G0 phase of cell cycle after 48hrs of *ex vivo* culture of E14.5 *IκBα* WT or KO FL lin- cells treated with 10uM of Ro-41 or DMSO.

Supplementary Figure S9

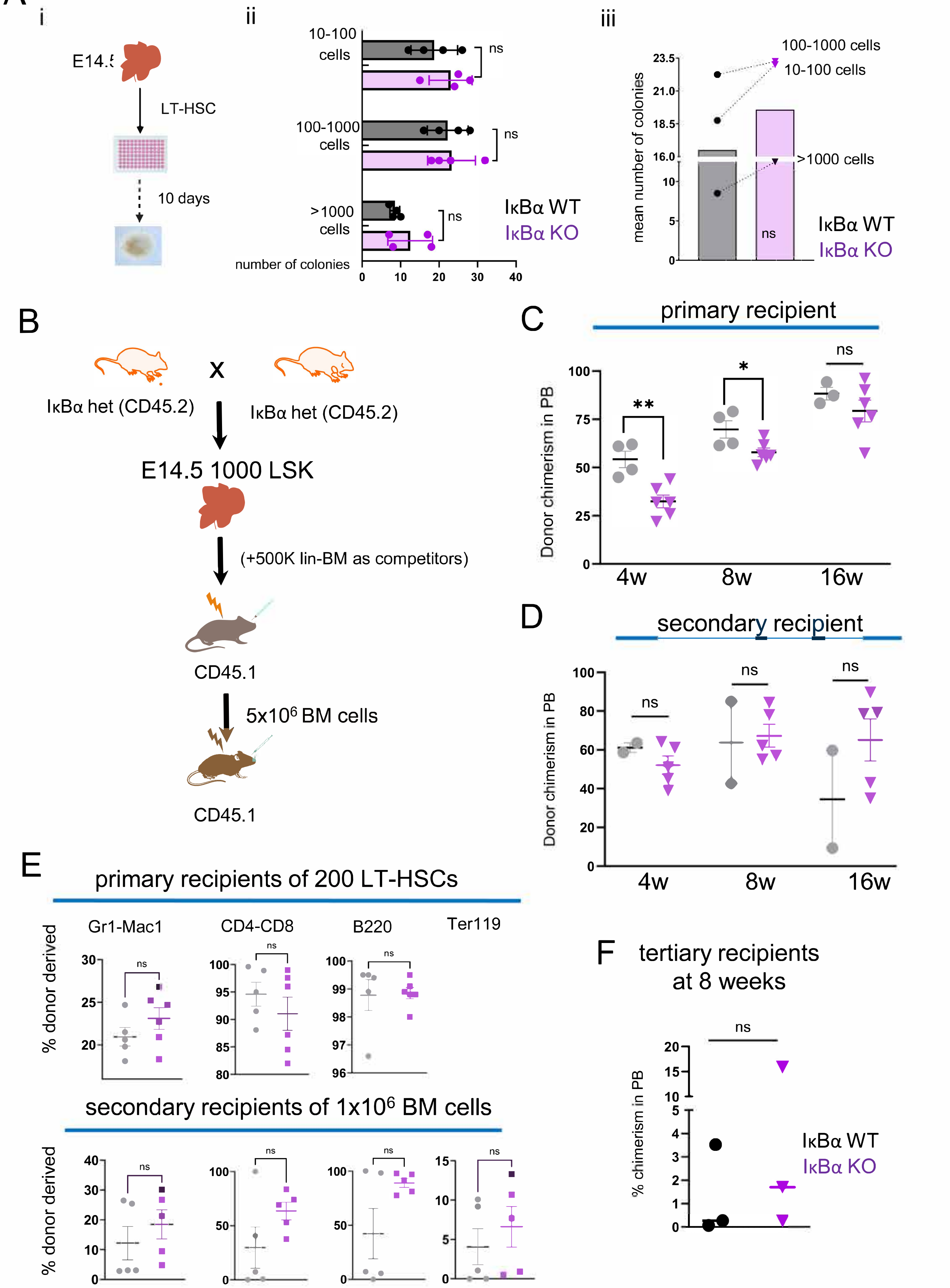

Suppl Figure S9: *IκBα* KO LSK are activated with a delay in transplantation assay

(**A**) **i.** scheme of experimental procedure. LT-HSCs from *IκBα* WT or KO E14.5 fetal liver are deposited as single cells into 96 well plates and colony number and size are scored 10 days later. **ii.** Bar chart with the number of hematopoietic colonies obtained from LT-HSC deposited as single cell after 10 days in culture from *IκBα* WT or KO E14.5 FL. n= 4 embryos per genotype in 2 independent experiments. Statistical test: unpaired one-tailed t-test with Welch correction (ns p-value > 0.05). The horizontal bars indicate mean values and error bars refer to +/- standard deviation. **iii.** dot plot with the mean values of colonies of the 4 replicates of the colonies shown in iii, separated colony size. (**B**) Scheme of the transplantation experiment. 1000 LSK cells were FACS purified from E14.5 *IκBα* WT and KO fetal liver (CD45.2) and transplanted serially into lethally irradiated donors (CD45.1). (**C**) Scatter plot with the percentage of donor chimerism (CD45.2) in the peripheral blood of primary recipient (CD45.1) in 4-week intervals. Recipients of 1000 purified LSK cells from pools of E14.5 fetal liver with n=4 WT and n=6 KO recipients. Statistical test: unpaired one-tailed Mann Whitney U test for all pairwise comparisons (** p-value < 0.01, ns p-value>0.05). The horizontal bars indicate mean values and error bars refer to +/- standard deviation. (**D**) Scatter plot with the percentage of donor chimerism (CD45.2) in the peripheral blood of secondary recipient (CD45.1) in 4-week intervals. Each secondary recipient received $1x10^6$ nucleated bone marrow cells from the primary recipients. n= 7 recipients. Statistical test: unpaired one-tailed Mann Whitney U test for all pairwise comparisons (** p-value < 0.01, ns p-value>0.05). The horizontal bars indicate mean values and error bars refer to +/- standard deviation. (**E**) Scatter plots with the percentages of donor chimerism (CD45.2) in the different blood lineages. Top: primary recipient of 200 LT-HSCs (n=5 WT, n=6 KO), bottom: secondary recipients that received $1x10^6$ nucleated bone marrow cells from the primary recipients at the terminal stage, i.e., 4 months after transplantation (n=5 WT, n=5 KO). Peripheral blood was analyzed for the lymphoid (CD4/8 and B220) and erythroid (Ter119), and bone marrow for myeloid (Gr1/Mac1) lineages. Statistical test: unpaired two-tailed Mann Whitney U test for all pairwise comparisons (ns p-value>0.05). The horizontal bars indicate mean values and error bars refer to +/- standard deviation. (**F**) Bar chart with the percentage of donor chimerism (CD45.2) in the peripheral blood of tertiary recipient (CD45.1) at 8 week. Each tertiary recipient received $1x10^6$ nucleated bone marrow cells from the secondary recipients. n= 6 recipients. Statistical test: unpaired one-tailed Mann Whitney U test (ns p-value>0.05). The horizontal bars indicate mean values. Source data are provided as a Source Data file. Panels A and B were created with BioRender.com released under a Creative Commons Attribution-NonCommercial-NoDerivs 4.0 International license.
