# Supplementary material for: IκBα controls dormancy in hematopoietic stem cells via retinoic acid during embryonic development
Source: Nat Commun. 2024 Jun 1;15:4673. doi: 10.1038/s41467-024-48854-5 (PMC11144194; doi:10.1038/s41467-024-48854-5)
Supplement: Supplementary file 3 — Description of Additional Supplementary Files [file 41467_2024_48854_MOESM3_ESM.pdf]

Description of Additional Supplementary Files

File Name: Supplementary Data 1
Description: table related to single cell data analysed from Zhou et al, 2016 in Figure 1.

File Name: Supplementary Data 2
Description: DEG of different clusters determined in the scRNA seq of LSK cells between Iκbα WT and KO.

File Name: Supplementary Data 3
Description: DEG, GSEA, and ChEA used for analysing bulk RNA seq of E14.5 FL LT-HSCs from Iκbα WT, Het and KO.

File Name: Supplementary Data 4
Description: table with peak calling and determination of consensus and unique peaks in E14.5 FL LT-HSC of Iκbα WT and KO for the histone mark H3K27me3.

File Name: Supplementary Data 5
Description: List of all antibody used in this study with their clone, supplier, catalog number, LOT and dilution factor.
