# Supplementary material for: IκBα controls dormancy in hematopoietic stem cells via retinoic acid during embryonic development
Source: Nat Commun. 2024 Jun 1;15:4673. doi: 10.1038/s41467-024-48854-5 (PMC11144194; doi:10.1038/s41467-024-48854-5)
Supplement: Supplementary file 9 — Reporting Summary [file 41467_2024_48854_MOESM9_ESM.pdf]

nature portfolio

Corresponding author(s): Anna Bigas/Roshana Thambyrajah

Last updated by author(s): Apr 17, 2024

Reporting Summary

Nature Portfolio wishes to improve the reproducibility of the work that we publish. This form provides structure for consistency and transparency in reporting. For further information on Nature Portfolio policies, see our Editorial Policies and the Editorial Policy Checklist.

Statistics

For all statistical analyses, confirm that the following items are present in the figure legend, table legend, main text, or Methods section.

n/a	Confirmed	
☐	☒	The exact sample size (*n*) for each experimental group/condition, given as a discrete number and unit of measurement
☐	☒	A statement on whether measurements were taken from distinct samples or whether the same sample was measured repeatedly
☐	☒	The statistical test(s) used AND whether they are one- or two-sided<br>*Only common tests should be described solely by name; describe more complex techniques in the Methods section.*
☐	☒	A description of all covariates tested
☐	☒	A description of any assumptions or corrections, such as tests of normality and adjustment for multiple comparisons
☐	☒	A full description of the statistical parameters including central tendency (e.g. means) or other basic estimates (e.g. regression coefficient) AND variation (e.g. standard deviation) or associated estimates of uncertainty (e.g. confidence intervals)
☐	☒	For null hypothesis testing, the test statistic (e.g. *F*, *t*, *r*) with confidence intervals, effect sizes, degrees of freedom and *P* value noted<br>*Give P values as exact values whenever suitable.*
☒	☐	For Bayesian analysis, information on the choice of priors and Markov chain Monte Carlo settings
☒	☐	For hierarchical and complex designs, identification of the appropriate level for tests and full reporting of outcomes
☒	☐	Estimates of effect sizes (e.g. Cohen's *d*, Pearson's *r*), indicating how they were calculated

Our web collection on statistics for biologists contains articles on many of the points above.

Software and code

Policy information about availability of computer code

Data collection	- Sorts were performed on AriaII or Influx instrument (BD Bioscience)<br>- FACS analysis was performed on Fortessa Instrument from BD Bioscience<br>- FACS data was collected in a Diva (v.6.1.3) or SpectralFlow (v3.0.3) software<br>- Imaging data was collected with Leica software (v.5.1)<br>- qPCRs were performed with a Lighcycler II instrument (v.1.5)<br><br>- scRNAseq data was collected from an Illumina NovaSeq6000 sequencer platform.<br>- RNAseq data was collected from an Illumina HiSeq2000 sequencer platform.<br>- CU&Tag data was collected from following sequencer platforms:<br>(i) HiSeq2500 for H3K27me3 related samples and IkBa AGM 1st replicate<br>(ii) NovaSeq6000 for IkBa LT-HSC 2nd replicate<br>iii) NextSeq500 for IkBa LT-HSC 1st replicate and IkBa AGM 2nd replicate
Data analysis	Regarding scRNAseq public dataset from Zhou et al. 2016, data analysis included:<br>- STAR aligner tool (v2.7.9a)<br>- R software environment (v4.1.0)<br>- DropletUtils package (v1.12.1)<br>- Seurat package (v4.0.2)<br>- UCell package (v2.0.1)

- ggsignif package (v0.6.4)
- FACS data was collected in a Diva (v.6.1.3) software

Scripts that have been used to process this public dataset in are deposited in a Github repository: https://github.com/zakiF/PublishedPapers/tree/master/Nat_comms_Ikba.

Regarding E14.5 LSK scRNAseq generated dataset, data analysis included:
- CellRanger (v6.0.0)
- R software environment (v4.1.3)
- Seurat package (v4.1.1)
- Monocle2

Regarding RNAseq data analysis:
- FASTQC tool (v0.11.9)
- Trimgalore (v0.6.6)
- STAR aligner tool (v2.7.8)
- R software environment (v4.2.1)
- DESeq2 Bioconductor R package (v1.38.3)
- removeBatchEffect from limma Bioconductor R package (v3.54.2)
- fgsea package (v1.24.0)
- ggplot2 package (v3.4.1)
- complexHeatmap Bioconductor R package (v2.14.0)
- EnhancedVolcano Bioconductor R package (v1.16.0)

Regarding CUT&Tag data analysis:
- FASTQC tool (v0.11.9)
- Trimgalore (v0.6.6)
- Bowtie2 (v2.4.4)
- Samtools (v1.15)
- SEACR (v1.3)
- deepTools (v3.5.1)
- R software environment (v4.2.1)
- ChIPseeker Bioconductor R package (v1.10.3)
- ChIPpeakAnno Bioconductor R package (v3.32.0)
- MEMEsuite CLI (v5.5.2)

Scripts that have been used to process the in house bulk RNA-seq and CUT&Tag data are deposited in Github repository: https://github.com/BigaSpinosaLab/HSC_dormancy_Ikba_via_retinoic_acid .

Other sofware used:
- GraphPad Prism 8.0.2
- FlowJO v10.7.2 CL
- Imaris (v10.1)

For manuscripts utilizing custom algorithms or software that are central to the research but not yet described in published literature, software must be made available to editors and reviewers. We strongly encourage code deposition in a community repository (e.g. GitHub). See the Nature Portfolio guidelines for submitting code & software for further information.

Data

Policy information about availability of data

All manuscripts must include a data availability statement. This statement should provide the following information, where applicable:
- Accession codes, unique identifiers, or web links for publicly available datasets
- A description of any restrictions on data availability
- For clinical datasets or third party data, please ensure that the statement adheres to our policy

Single cell RNA-seq, bulk RNA-seq and CUT&Tag data generated in this study have been deposited in NCBI Gene Expression Omnibus (GEO) repository under GEO SuperSeries accession no. GSE188525[https://www.ncbi.nlm.nih.gov/geo/query/acc.cgi?acc=GSE188525], composed in respective SubSeries
GSE214699 [https://www.ncbi.nlm.nih.gov/geo/query/acc.cgi?acc=GSE214699],
GSE188523 [https://www.ncbi.nlm.nih.gov/geo/query/acc.cgi?acc=GSE188523] and
GSE188524 [https://www.ncbi.nlm.nih.gov/geo/query/acc.cgi?acc=GSE188524].
Additionally, single cell RNA-seq data from Zhou et al was downloaded from GEO with accession number GSE67120 [https://www.ncbi.nlm.nih.gov/geo/query/acc.cgi?acc=GSE67120].

Research involving human participants, their data, or biological material

Policy information about studies with human participants or human data. See also policy information about sex, gender (identity/presentation), and sexual orientation and race, ethnicity and racism.

Reporting on sex and gender	n/a
Reporting on race, ethnicity, or other socially relevant groupings	n/a
Population characteristics	n/a
Recruitment	n/a
Ethics oversight	n/a

Note that full information on the approval of the study protocol must also be provided in the manuscript.

Field-specific reporting

Please select the one below that is the best fit for your research. If you are not sure, read the appropriate sections before making your selection.

[x] Life sciences [ ] Behavioural & social sciences [ ] Ecological, evolutionary & environmental sciences

For a reference copy of the document with all sections, see nature.com/documents/nr-reporting-summary-flat.pdf

Life sciences study design

All studies must disclose on these points even when the disclosure is negative.

Sample size	We used mouse embryonic (El0-Ell.5) trunk tissues, E14.5 fetal liver and fetal livers/bone marrow of newborn mice (age 5/6 days after birth). Sample size was estimated based on the number of embryos and independent replicates needed for statistical tests. At least 3 independent embryos/samples were assessed for each experimental procedure to determine the conclusion. Whenever there was a high biological variation, additional samples were added to assess if statistical significance was possible or not.
Data exclusions	We excluded one LT-HSC RNA seq sample (KO) due to low quality of the sample.
Replication	Each reported experiment was performed at least in two independent experiments, ie repeating the entire protocol/method. In each experiment, at least 3 individual embryos were sampled. We also used different instruments (BD Fortessa, Cytek Aurora, BD Ariail and BDInflux) and methods (FACS analysers, C&T, transplantation assays and PLA assays) to validate and cross-reference our findings. All replications were successful.
Randomization	We did not selectively pick individual embryos for any of the experiments. In fact, we used Ikba WT, Het and KO from the same litter whenever possible in each of the independent experiments.
Blinding	Since we need to genotype the embryos first to know which of them have the desired genotype (only 1 in 4 are WT or KO), it was not possible to perform the experiments blind.

Reporting for specific materials, systems and methods

We require information from authors about some types of materials, experimental systems and methods used in many studies. Here, indicate whether each material, system or method listed is relevant to your study. If you are not sure if a list item applies to your research, read the appropriate section before selecting a response.

Materials & experimental systems

n/a	Involved in the study	
[ ]	[x]	Antibodies
[x]	[ ]	Eukaryotic cell lines
[x]	[ ]	Palaeontology and archaeology
[ ]	[x]	Animals and other organisms
[x]	[ ]	Clinical data
[x]	[ ]	Dual use research of concern
[x]	[ ]	Plants

Methods

n/a	Involved in the study	
[ ]	[x]	ChIP-seq
[ ]	[x]	Flow cytometry
[x]	[ ]	MRI-based neuroimaging

Antibodies

Antibodies used

Name clone cat# Lot Supplier concentration
Streptavidin PerCP-Cy5.5 n/a 45-4317-82 2198664 eBioscience 1/200
c-KIT–APC–eFluor780 2B8 47-1171-82 2373373 ebioscience 1/200
Sca1_FITC E13-161.7 553335 1187894 BiophaMingen 1/200
CD48 PE-Cy7 HM48-1 560731 3115600 BD Pharmingen, BrdU kit 1/400
CD150 (SLAM) Pe-Cy5 TC15-12F12.2 115912 B239441 BioLegend 1/400
CD150 (SLAM) PE TC15-12F12.2 115904 B338816 BioLegend 1/400
CD150(SLAM)-APC TC15-12F12.2 115910 B317517 eBioscience 1/200
BrdU APC n/a ab220075 2290051 BD Pharmingen, BrdU kit 1/200
Ki67 eflour 700 B56 561277 1154059 BD Bioscience 1/200

CD31 Alexa 647 Mec13.3 #102515 B290400 BioLegend 1/200
c-KIT–APC–eFluor780 2B8 47-1172-82 2373373 ebioscience 1/200
CD45 PerCP5.5 3D-F11 45-0451-82 2622634 Invitrogen 1/200
CD201 (PROCR/EPCR) eFL 710 eBio156D 46-2012-80 2158264 Invitrogen 1/200
CD201 (PROCR/EPCR) eFL 780 eBio1560 47-2012-82 2518488 Invitrogen 1/200
Sca1_FITC E13-161.7 553335 1187894 BiophaMingen 1/200
CD45.1 APC-eFluor 780 A20 47-0453-82 2526336 eBioscience 1/200
CD45.2 PerCP-Cy5.5 (RUO) 104 552950 2094204 BD Pharmingen 1/200
Gr1-APC RB6-8C5 17-5931-82 1976250 eBioscience 1/200
Mac1-FITC M1/70 557396 2049680 PharMingen 1/200
CD4-FITC L3T4 553046 57932 PharMingen 1/200
CD8-PE Ly-2 553032 7283861 PharMingen 1/400
B220 Pe-Cy7 RA3-6B2 552772 7188819 BD Pharmingen 1/400
Ter119-APC TER-119 557909 5210888 BD Pharmingen 1/200

Primary antibodies

Name clone cat# Lot Supplier concentration
goat anti IκB-α C-21 discontinued (SC-371-G) G2816 Santa Cruz 1/200
IkB-a Phospho ser 32/36 5A5 9246 23 Cell signaling 1/200
p65 polyclonal ab16502 GR3293262-1 abcam 1/200
Anti-NF-kB p65 phospho S536 93H1 3033 16 Cell signaling 1/200
biotinylated rat anti-mouse CD31 clone MEC13.3 553371 5016615 BD Bioscience 1/200

Proximity ligation assay

Name clone cat# Lot Supplier concentration
goat anti IκB-α C-21 discontinued (SC-371-G) G2816 Santa Cruz 1/100
rabbit anti-EZH2 #4905 polyclonal unknown cell signaling 1/100

goat anti DLL4 polyclonal AF1389 JPI0117041 R&D novus 1/200

antibodies_cut and tag
Name clone cat# Lot Supplier concentration
goat anti IκB-α C-21 discontinued (SC-371-G) G2816 Santa Cruz 1/100
Tri-Methyl-Histone H3 (Lys27) C36B11 #9733 19 cell signaling 1/100
guinea pig α-rabbit antibody polyclonal ABIN101961 43586 antibodiesonline 1/100
rabbit anti IκBα P36 recombinant ab133462 GR3378494-16 abcam 1/100

Secondary antibodies

Alexa Fluor 647 goat anti-rat goat anti-rat A-21247 1182663 Life Technologies 1/400
Alexa fluor 488 donkey anti-goat donkey anti-goat A11055 2059218 Molecular probes 1/400
Alexa Fluor 488 donkey anti-rabbit donkey anti-rabbit A212O6 2541645 Molecular probes 1/400
Alexa Fluor 546 donkey anti-rabbit donkey anti-rabbit A10040 2273718 Life Technologies 1/400

Validation

Established antibodies (FACS and santibodies for IHC) were validated in house by FACS with single staining and FMO controls. In IHC and PLA, antibodies were tested by comparing the signal to secondary only control, or by comparing the expression pattern with published data sets. pIkba Ser32/36 and Ikba (total) antibodies were tested for specificity in Ikba KO animals in our laboratory.

Animals and other research organisms

Policy information about studies involving animals; ARRIVE guidelines recommended for reporting animal research, and Sex and Gender in Research

Laboratory animals	Mice born in the Barrier area are socially housed, up to four male and five female mice, in 1145T (Tecniplast) cages in individually ventilated cages. Autoclaved black poplar shavings (Souralit) is used as bedding, and irradiated tissues as nesting material. Autoclaved cardboard cylinders are added to cages with mating pairs and individually-housed mice as additional environmental enrichment. Once a week mating pairs and once evry two weeks the rest of socially housed mice, together with the nesting material, are transferred to clean cages. Mice have ad libitum access to autoclaved water and irradiated diet (J. Rettenmaier & Söhne GmbH + CO KG ; RM3 for breeding pairs and young mice until nine weeks old, and RM1 as maintenance diet after nine weeks of age). Rooms are maintained under standard environmental conditions (humidity: 40–60%; temperature: 20–24℃) and a 12h light/dark cycle (lights on at 08:00h). Animal care and use programme is approved by PRBB-Ethics Committee and accredited by AAALAC International, following European (2010/63/UE) and Spanish (RD 53/2013) regulations.<br>The CDI-IGS wild type strain, thr Gfil:tomato (Thambyrajah et al., 2016) and Iκ Bα targeted knock out mouse line (Nfkbia tmlBal, Jackson Laboratories, stock #002850) were used in this study. For time matings, Gfil:tomato, Ikba het or CDI WT females were mated to Gfi:tomato, Ikba het or CDI WT males. In transplantation assays, C57BL/6 (CD45.1) males and females between the ages of 8-12 weeks were subject to irradiation and were housed as described here.
Wild animals	Wild animals were not used in this study.
Reporting on sex	In general, this does not apply to this study since embryos were collected at mid-gastation (E10.5-E16.5) and newborn (P5/6). In fact, no sex bias was introduced in this study since the gender was not determined. However, de did detect the gender of the embryo in our RNA seq data and we have reported this in the figure (Suupl Figure S4F).
Field-collected samples	We did not use any field-collected animals in this study.
Ethics oversight	Animal Care Committee of the Parc de Recerca Biomedica de Barcelona, license number 9309 approved by the Generalitat de Catalunya.

Note that full information on the approval of the study protocol must also be provided in the manuscript.

Plants

Seed stocks	n/a
Novel plant genotypes	n/a
Authentication	n/a

ChIP-seq

Data deposition

☒ Confirm that both raw and final processed data have been deposited in a public database such as GEO.

☒ Confirm that you have deposited or provided access to graph files (e.g. BED files) for the called peaks.

Data access links<br>*May remain private before publication.*	https://www.ncbi.nlm.nih.gov/geo/query/acc.cgi?acc=GSE188524
Files in database submission	Regarding Ikba Ab samples, raw (fastq files) and processed data (bigwig files, no peak calling conducted) are available, specifically for the bigwig files:<br><br>a) CD31+ replicates:<br>Ikba_AAB_E11.5_Ikba_AGM_1.RPGC.bw<br>E11-IK_S8_E11.5_Ikba_AGM_2.RPGC.bw<br><br>b) LT-HSCs replicates:<br>E14.5_Ikba_LT-HSC_1.RPGC.bw<br>RT_4_lib_16752AAE_E14.5_Ikba_LT-HSC_2.RPGC.bw

No peak calling was conducted for these samples.

Regarding H3K27me3 Ikba WT and KO samples, raw (fastq files) and processed data (bigwig files and BED files with called peaks) are available, specifically for the:

a) BigWig files:
H3K27me3_Ikba_KO_LT-HSC_1.RPGC.final.bw
H3K27me3_Ikba_KO_LT-HSC_2.RPGC.final.bw
H3K27me3_Ikba_KO_LT-HSC_3.RPGC.final.bw
H3K27me3_Ikba_WT_LT-HSC_1.RPGC.final.bw
H3K27me3_Ikba_WT_LT-HSC_2.RPGC.final.bw
H3K27me3_Ikba_WT_LT-HSC_3.RPGC.final.bw

b) BED files:
Ikba_KO_LT-HSC_1.woDups.top0.5.stringent.filtered_blacklisted.bed
Ikba_KO_LT-HSC_2.woDups.top0.5.stringent.filtered_blacklisted.bed
Ikba_KO_LT-HSC_3.woDups.top0.5.stringent.filtered_blacklisted.bed
Ikba_WT_LT-HSC_1.woDups.top0.5.stringent.filtered_blacklisted.bed
Ikba_WT_LT-HSC_2.woDups.top0.5.stringent.filtered_blacklisted.bed
Ikba_WT_LT-HSC_3.woDups.top0.5.stringent.filtered_blacklisted.bed

Genome browser session
(e.g. UCSC)

No longer applicable

Methodology

Replicates

For Cut and Tag assays, either CD31+ (25.000-30.000 cells) from E11.5 AGMs or LT-HSCs from E14.5 fetal livers (3.000-4.000 cells of IκBα WT or KO) were FACS sorted and used for cut and tag assay of IκBα and H3K27me3, respectively. We followed the Bench top CUT&Tag V.3 (https://www.protocols.io/view/bench-top-cut-amp-tag-bcuhiwt6), an updated version from Kaya-Okur et al, 2019 with their recommended antibody for H3K27me3 (for details, please see Suppl table T5) and loaded Tn5 from epicypher. After the assay, the samples were quantified and validated using a bio analyzer and subsequently paired-end sequenced. We procuded three independent replicates for H3K27me3 experiment. For Ikba (CD31+) we have two independent replicates and for Ikba (LT-HSCs) we have two replicates from 2 different antibodies.

Sequencing depth

Regarding CUT&Tag Ikba Ab samples, for the:

a) CD31+ replicates:
E11.5_Ikba_CD31_1: Paired-end 50bp read length. Total number of raw reads: 2417649 reads. Total number of reads after trimming: 2415271 reads. Uniquely mapped reads: 1434658 reads (59.40% referred to trimmed reads)
E11.5_Ikba_CD31_2: Paired-end 75bp read length. Total number of raw reads: 1640552 reads. Total number of reads after trimming: 1640516 reads. Uniquely mapped reads: 978565 reads (59.65% referred to trimmed reads)

b) LT-HSC replicates:
E14.5_Ikba_LT-HSC_1: Paired-end 75bp read length. Total number of raw reads: 19916107 reads. Total number of reads after trimming: 19904287 reads. Uniquely mapped reads: 12309970 reads (61.85% referred to trimmed reads)
E14.5_Ikba_LT-HSC_2: Paired-end 150bp read length. Total number of raw reads: 8146658 reads. Total number of reads after trimming: 8142725 reads. Uniquely mapped reads: 4434891 reads (54.46% referred to trimmed reads)

Regarding CUT&Tag H3K27me3 Ikba WT and KO samples, for the:

a) Ikba WT replicates:
Ikba_WT_LT-HSC_H3K27me3_1: Paired-end 75bp read length. Total number of raw reads: 12587294 reads. Total number of reads after trimming: 12580012 reads. Uniquely mapped reads: 8686501 reads (69.05%)
Ikba_WT_LT-HSC_H3K27me3_2: Paired-end 75bp read length. Total number of raw reads: 16871293 reads. Total number of reads after trimming: 16861431 reads. Uniquely mapped reads: 11931720 reads (70.76%)
Ikba_WT_LT-HSC_H3K27me3_3: Paired-end 50bp read length. Total number of raw reads: 5603084 reads. Total number of reads after trimming: 5597316 reads. Uniquely mapped reads: 3787612 reads (67.67%)

b) Ikba KO replicates:
Ikba_KO_LT-HSC_H3K27me3_1: Paired-end 75bp read length. Total number of raw reads: 10397542 reads. Total number of reads after trimming: 10391835 reads. Uniquely mapped reads: 7194263 reads(69.23%)
Ikba_KO_LT-HSC_H3K27me3_2: Paired-end 75bp read length. Total number of raw reads: 13278732 reads. Total number of reads after trimming: 13270773 reads. Uniquely mapped reads: 9470148 reads (71.36%)
Ikba_KO_LT-HSC_H3K27me3_3: Paired-end 50bp read length. Total number of raw reads: 6383260 reads. Total number of reads after trimming: 6375549 reads. Uniquely mapped reads: 4433548 reads (69.54%)

Antibodies

antibodies_cut and tag

Name clone Supplier concentration

goat anti IκB-α C-21 Santa Cruz 1/100
Tri-Methyl-Histone H3 (Lys27) C36B11 cell signaling 1/100
guinea pig α-rabbit antibody ABIN101961 antibodiesonline 1/100
rabbit anti IκBα P36 recombinant ab133462 1/100

Peak calling parameters

All CUT&Tag samples were mapped with Bowtie2 aligner tool and SAMtools was used for reads quality filtering (remove those reads with MAPQ < 20 or unmapped). Additionally multimappers were excluded (with grep). The specific executed command was:

```
bowtie2  -x $INDEX -1 $READ1 -2 $READ2 --no-unal --no-mixed --no-discordant --dovetail --very-sensitive-local \
2> $SAMPLE.align.stats.txt  \
| samtools view -F 4 -h -q 20 \
| grep -v 'XS:i:' \
| samtools sort -O BAM \
| tee $SAMPLE.sorted.unique.bam | samtools index - $SAMPLE.sorted.unique.bam.bai
```

where $SAMPLE refers to a specific sample name, $READ1 and $READ2 refer to corresponding fastq files (forward and reverse) for that specific sample and, $INDEX refers to the genome assembly computed index for Bowtie2 (mm10 Ensembl release 102).

Peak calling was only executed for H3K27me3 samples . For this purpose, SEACR too (v1.3)l was used. Tool script was downloaded from https://github.com/FredHutch/SEACR and executed with following parameters: stringent mode, without IgG sample and selecting the top 0.5% peaks.

Data quality

Data quality was first ensured by initial inspection of FASTQ files (with FASTQC) to detect any possible anomaly in the generated data. After mapping, percentages of unique mapped reads were inspected to assess whether a particular samples presented low quality in terms of quality mapping. In fact, those reads with mapping quality below MAPQ <20 were excluded. In terms of peak calling (only applicable for H3K27me3 Ikba WT and KO samples), SEACR tool was executed in 'stringent' mode instead of 'relaxed' mode which represents a more conservative peaks detection. Moreover, only the top 0.5% of peaks were kept for downstream analysis. Overall, three replicates were used per condition, and although, individual peaks are reported, a consensus peakset among the three replicates (in WT or KO condition) is the one used for further analysis (i.e. motif discovery).

Regarding the number of called peaks per sample:

a) Ikba WT replicates:
Ikba_WT_LT-HSC_H3K27me3_1: 4160 peaks
Ikba_WT_LT-HSC_H3K27me3_2: 6013 peaks
Ikba_WT_LT-HSC_H3K27me3_3: 1047 peaks

b) Ikba KO replicates:
Ikba_KO_LT-HSC_H3K27me3_1: 4904 peaks
Ikba_KO_LT-HSC_H3K27me3_2: 5157 peaks
Ikba_KO_LT-HSC_H3K27me3_3: 2325 peaks

Software

All software used to collect and analyze the data are standard tools widely used for this type of data. No custom or special code has been required for executing them. Nevertheless, scripts for analyzing CUT&Tag data are publicly available in the following GitHub repository: https://github.com/BigaSpinosaLab/HSC_dormancy_Ikba_via_retinoic_acid

Flow Cytometry

Plots

Confirm that:

- [x] The axis labels state the marker and fluorochrome used (e.g. CD4-FITC).
- [x] The axis scales are clearly visible. Include numbers along axes only for bottom left plot of group (a 'group' is an analysis of identical markers).
- [x] All plots are contour plots with outliers or pseudocolor plots.
- [x] A numerical value for number of cells or percentage (with statistics) is provided.

Methodology

Sample preparation

AGMs
AGMs of E10- E11.5 embryos were dissected in PBS with 7% fetal calf serum (FBS) and penicillin/streptomycin (100 U/mL). Single cell suspensions were generated by incubating the tissues for 20-30 minutes in 500 ul of 1mg/ml of Collagenase/ Dispase (Roche cat# 10269638001) before mechanical dissociation with a syringe and needle. The resulting single cell suspension was used for antibody staining.

fetal liver and bone marrow samples

Mouse bone marrow cells were obtained by extracting the femur and tibia bones and subsequent crushing of the bones in cold PBS with 0.5% FBS Buffer using a mortar and pestle. Cells were then labeled with a cocktail of antibodies against lineage markers (BD Bioscience, cat# 559971) before conjugation to anti Biotin microBeads (Miltenyi Biotech, cat #130-090-485). CD11b was not included in the lineage depletion mix. Labeled cells were depleted using magnetic columns (Miltenyi Biotech), leaving behind isolated unlabeled Lin– cells immediately ready for downstream experiments. For FL, the tissue was harvested from newborn or E14.5 embryos and the dissected liver was crushed with the end of a 1ml syringe through a 40um cell strainer into IMDM +10% FBS. the resulting single cell suspension was used for antibody staining.

Instrument

FACS analyzers: BD Fortessa or Cytek Aurora
iFACS sorters: Ariall or BD Influx (BD Biosciences)

Software

FACS plots were generated using FACS Diva software or Flowio V10

Cell population abundance

FACS sorters were used in the "high purity" setting. Unfortunately, due to the small number of cells of the samples, we could not run post-sort purity test.

Gating strategy

First, using FSC-A and SSC-A we excluded cells debris. In the second step, FSC-W and FSC-A were used to gate on single cells, followed by exclusion of dead cells with DAPI staining. In the first experiment of each staining Florescence minus one controls were used to establish the cut off lines for each gate. We also cross-examined how the staining of a given positive population behaved within a known negative control population within the same sample. Finally, we also checked and compared our gates and staining efficiency with published data sets whenever possible. Frequency of a population from the previous (indicated) gate are shown in each FACS plot.

☒ Tick this box to confirm that a figure exemplifying the gating strategy is provided in the Supplementary Information.
